# An active fixation quadripolar left ventricular lead for cardiac resynchronization therapy with reduced postoperative complication rates

**DOI:** 10.1111/jce.15346

**Published:** 2022-01-11

**Authors:** Calum Robertson, Owen Duffey, Pok‐Tin Tang, Natalie Fairhurst, Cristiana Monteiro, Peregrine Green, Joanna Grogono, Mark Davies, Andrew Lewis, Rohan Wijesurendra, Julian Ormerod, James Gamble, Matthew Ginks, Kim Rajappan, Yaver Bashir, Tim R. Betts, Neil Herring

**Affiliations:** ^1^ Department of Cardiology Oxford Heart Centre, John Radcliffe Hospital, Oxford University Hospitals NHS Foundation Trust Oxford UK; ^2^ Department of Physiology Anatomy and Genetics, Burdon Sanderson Cardiac Science Centre University of Oxford Oxford UK; ^3^ Division of Cardiovascular Medicine, Radcliffe Department of Medicine University of Oxford Oxford UK

**Keywords:** active fixation, cardiac resynchronization therapy, complications, lead displacement, reintervention

## Abstract

**Background:**

The rate of left ventricular (LV) lead displacement after cardiac resynchronization therapy (CRT) remains high despite improvements in lead technology. In 2017, a novel quadripolar lead with active fixation technology became available in the UK.

**Methods:**

This was a retrospective, observational study analyzing device complications in 476 consecutive patients undergoing successful first‐time implantation of a CRT device at a tertiary center from 2017 to 2020.

**Results:**

Both active (*n* = 135) and passive fixation (*n* = 341) quadripolar leads had similar success rates for implantation (99.3% vs. 98.8%, *p* = 1.00), although the pacing threshold (0.89 [0.60–1.25] vs. 1.00 [0.70–1.60] V, *p* = .01) and lead impedance (632 [552–794] vs. 730 [636–862] Ohms, *p* < .0001) were significantly lower for the active fixation lead. Patients receiving an active fixation lead had a reduced incidence of lead displacement at 6 months (0.74% vs. 4.69%, *p* = .036). There was no significant difference in the rate of right atrial (RA) and right ventricular (RV) lead displacement between the two groups (RA: 1.48% vs. 1.17%, *p* = .68; RV: 2.22% vs. 1.76%, *p* = .72). Reprogramming the LV lead after displacement was unsuccessful in most cases (successful reprogramming: Active fix = 0/1, Passive fix = 1/16) therefore nearly all patients required a repeat procedure. As a result, the rate of intervention within 6 months for lead displacement was significantly lower when patients were implanted with the active fixation lead (0.74% vs. 4.40%, *p* = .049).

**Conclusion:**

The novel active fixation lead in our study has a lower incidence of lead displacement and re‐intervention compared to conventional quadripolar leads for CRT.

## 1 INTRODUCTION

Cardiac resynchronization therapy (CRT) has become the gold standard treatment for heart failure patients with severely reduced ejection fraction and left bundle branch block. Studies have consistently demonstrated an improvement in symptoms and left ventricular (LV) ejection fraction in the majority of patients, as well as a reduction in mortality after CRT insertion.^1^, ^2^ Nonetheless, CRT is limited by several complications, many of which relate to the insertion of the LV lead.^3^ The placement of a pacing lead within the coronary venous system for CRT can be technically challenging and subsequent LV lead displacement is a well‐recognized postprocedural complication.^4^ This typically results in an increase in pacing threshold and a loss of LV capture, thus negating the benefit of resynchronization therapy. LV lead displacement can also cause inadvertent stimulation of the phrenic nerve, leading to uncomfortable diaphragmatic excitation. Whilst the advent of quadripolar LV leads allows for re‐programming around phrenic nerve stimulation,^5^ if LV lead displacement results in loss of capture from all the available pacing vectors, a repeat procedure to replace the lead is required.

Standard LV leads rely on a passive fixation technique which involves advancing the lead tip distally into the target vessel. With quadripolar leads, this can allow for the lead to be positioned more apically for better stability, but with pacing from more basal electrodes closer to the site of the latest activation.^6^ Quadripolar leads are associated with more effective resynchronization and a reduction in heart failure hospitalization and mortality compared to bipolar leads.^5^, ^7^, ^8^, ^9^ In 2017, a novel quadripolar LV lead‐containing active fixation mechanism was made available in the UK. This lead has a specifically designed side‐helix which actively fixes the lead body to the target vessel. Initial experience with the lead has demonstrated a high rate of procedural insertion success (96.8%) and a low rate of LV lead‐related complications (2.3%).^10^ However, no study to date has made a direct comparison between this active fixation lead and conventional quadripolar LV leads. In this study, we use observational data from a large tertiary UK hospital to assess lead implantation success, pacing characteristics, and complication rates when compared to conventional passive fixation quadripolar LV leads, with a focus on the rate of lead displacement at 6 months and the need for invasive re‐intervention.

## 2 METHODS

### 2.1 Lead design and implantation

**Figure 1:**
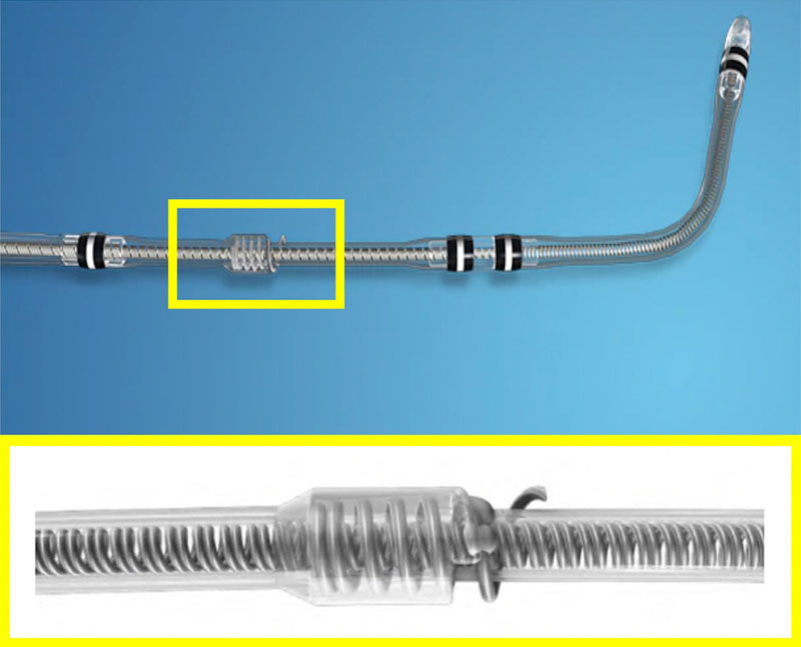
The Attain Stability Quad 4798 ‐ This left ventricular lead was released by Medtronic in the UK in 2017; shown here is the side‐helix for active fixation into the target vessel. Image reproduced with permission of Medtronic

The Medtronic Attain Stability Quad 4798 lead design is based on the Attain Performa family of steroid‐eluting quadripolar LV leads. A novel design feature of the lead is the positioning of an electrically inactive side helix between the third and fourth pacing electrodes (Figure 1). This side helix can be coiled into the vessel wall to actively fix the position of the lead. As is standard practice, the lead is inserted using a traditional over‐the‐wire technique into a tributary of the coronary sinus to pace the left ventricle. Once the operator is satisfied with the lead position, the fixation mechanism can be activated by clockwise rotation of the lead body. To preserve the active fixation mechanism during insertion, it is good practice to rotate the pacing lead anti‐clockwise whilst advancing it through the valve of the guide catheter sheath. Successful deployment of the active fixation mechanism can be confirmed by gently pushing and pulling on the pacing lead ‐ this should result in the opposite motion of the delivery catheter. If re‐positioning is required (or if the lead later requires extraction), anti‐clockwise rotation of the lead body releases the side helix from the vessel wall. The side helix is positioned 0.25 mm away from the lead body (the average distance between a vein and the nearest artery is 1 mm)^11^ leaving a fourfold safety margin for insertion. Furthermore, the helix incorporates a mechanical stop that activates after four rotations to prevent the lead from becoming over‐torqued.

### 2.2 Study design

We performed an observational study using registry data from a UK tertiary center to compare the complication rates for patients undergoing CRT insertion with the active fixation lead and those who received conventional passive fixation quadripolar leads. All patients gave written informed consent, and the study was locally approved by our institutional board (clinical improvement module 6935). It reflects the experience of genuine clinical practice in our center; every patient that underwent first‐time CRT insertion at the John Radcliffe Hospital (Oxford) with a quadripolar LV lead from 2017 to July 2020 was included in the study. Four hundred and seventy‐six patients were analyzed in total – 135 received the Medtronic Attain Stability Quad 4798, whereas 341 received conventional passive‐fixation quadripolar leads (St Jude Medical = 305, Medtronic = 27, and Boston Scientific = 9). All CRT devices were implanted according to guidelines published by the European Society of Cardiology.^12^

The decision to insert a passive or active fixation LV lead was made before the procedure by the implanting physician. Pre‐implantation characteristics such as patient demographics, ejection fraction, and electrophysiological parameters were collected from available clinical data. Procedural characteristics such as the position of the LV lead, and lead capture threshold were collected using our local catheter‐lab reporting system. Complete data sets were available for all parameters except for lead electrical characteristics (*Impedance*: Active fix = 121/135, Passive fix = 322/341; *Capture Threshold*: Active fix = 124/135, Passive fix = 322/341; *Pulse Width*: Active fix = 124/135, Passive fix = 322/341; *Energy threshold*: Active fix = 121/135, Passive fix = 322/341), and QRS duration (Active fix = 124/135, Passive fix = 322/341). Following implantation, all device programming was optimized before the patient left the operating theater with LV pacing including the lead electrode with the latest QLV^6^ (without phrenic nerve stimulation) and with optimization of V‐V delay to produce the narrowest QRS duration of the best morphology (with a positive R wave in lead V1) as per the Oxford protocol. The use of fixed, device‐optimized AV delays or fusion pacing algorithms (e.g., SyncAV™, AdaptivCRT™, and Smartdelay™) are at the discretion of the operator but are also optimized to produce the narrowest QRS duration of the best morphology. Multipoint™ or MultiSite™ pacing is not routinely programmed at implant in our institution but considered at 6 months in non‐responders.^13^ Electronic patient records were used to identify patients that experienced complications within 6 months, such as phrenic nerve stimulation and lead displacement. If there was an unacceptable rise in pacing thresholds, lack of ventricular capture, or new phrenic nerve stimulation, then patients would undergo a chest X‐ray to look for evidence of displacement. A lead displacement was defined as a change in the lead position as documented on chest radiography in two orthogonal planes associated with a change in pacing parameters from that observed at the implant. All complications were reviewed monthly at a morbidity and mortality meeting, and annually via further audit as approved by our institution.

### 2.3 Data analysis

All continuous nonparametric data is reported as a median and interquartile range. Analysis was performed using a Mann–Whitney U test. Categorical data were reported as *n* (%) and analyzed using a *χ* ^2^ test, except for where there was a small number of reported events, under which circumstances a Fisher's exact test was used. Statistical significance is accepted at *p* < .05.

## 3 RESULTS

### 3.1 Clinical characteristics

The clinical characteristics of the 476 patients who successfully received an LV lead are shown in Table 1. Patients receiving either an active or passive fixation quadripolar lead were well matched with respect to sex, heart failure etiology, and left ventricular ejection fraction. Furthermore, there were no significant differences between the two groups with respect to the QRS duration or underlying electrical rhythm. However, patients receiving the active fixation lead were on average slightly older.

**Table 1:** Clinical characteristics

	Active fix (*n* = 135)	Passive fix (*n* = 341)	*p* value
Age	78.2 [69.5–83.2]	74.2 [65.5–81.4]	**0.02**
Sex (number of females)	41 (30.4%)	83 (24.3%)	.18
Etiology			
Ischemic	51 (37.8%)	130 (38.1%)	
Non‐ischemic	84 (62.2%)	211 (61.9%)	.94
Left ventricular ejection fraction			
≤35%	96 (71.1%)	269 (78.9%)	
>35%	39 (28.9%)	72 (21.1%)	.07
Sinus rhythm at implant	104 (77.0%)	278 (81.5%)	.27
Mobitz II/CHB	6 (4.4%)	19 (5.57%)	.82
QRS duration pre‐implant (ms)	160 [150–180]	160 [144–180]	.29

*Note*: Data from 476 patients undergoing first‐time cardiac resynchronization therapy device implantation with either a conventional passive fixation quadripolar left ventricular lead or the active fixation lead.

Abbreviation: CHB, complete heart block.

### 3.2 Procedural characteristics

Procedural success in inserting an LV lead was similarly high regardless of the type of LV lead that was selected (Active fixation = 135/136 (99.3%); Passive fixation = 341/345 (98.8%), *p* = 1.00). For successful lead insertions, the procedural characteristics for each implantation are shown in Table 2. A similar proportion of active fixation CRT procedures incorporated a defibrillator when compared with the passive fixation group, although a slightly higher proportion were de novo insertions rather than an upgrade of an existing pacemaker. LV lead pulse width (*t*, seconds) was the same for both groups. Whilst the capture threshold (*V*) was significantly lower with the active fixation lead, the impedance (*I*) was similarly lower, therefore there were no significant differences in the threshold energy between the two groups. Documentation of the final LV lead position obtained using our cath‐lab reporting system demonstrated that there was no significant difference in the anatomical positioning of active and passive fixation LV leads.

**Table 2:** Procedural characteristics

	Active fix (*n* = 135)	Passive fix (*n* = 341)	*p* value
First CRT procedure			
De novo insertions	110 (81.5%)	244 (71.6%)	
Pacemaker upgrade	25 (18.5%)	97 (28.4%)	**.03**
Device type			
CRT‐P	85 (63.0%)	198 (58.1%)	
CRT‐D	50 (37.0%)	143 (41.9%)	.33
Lead manufacturer			
Medtronic	135	27	
St Jude Medical		305	
Boston Scientific		9	
LV lead characteristics			
Impedance (Ohms)	632 [552–794]	730 [636–862]	<**.0001**
Capture threshold (V)	0.89 [0.60–1.25]	1.00 [0.70–1.60]	**.01**
Pulse width (ms)	0.40 [0.40–0.50]	0.50 [0.40–0.50]	.09
Energy threshold (microJ)	0.57 [0.30–1.26]	0.70 [0.30–1.80]	.13
Position of LV lead			
Posterolateral	55 (40.7%)	133 (39.0%)	
Lateral	66 (48.9%)	154 (45.2%)	
Anterolateral	10 (7.4%)	40 (11.7%)	
Middle cardiac	4 (2.9%)	14 (4.11%)	.49

*Note*: Data from 476 patients undergoing first‐time cardiac resynchronization therapy device implantation with either a conventional passive fixation quadripolar LV lead or the active fixation lead.

Abbreviations: CRT, cardiac resynchronization therapy; LV, left ventricular.

### 3.3 Complications

**Figure 2:**
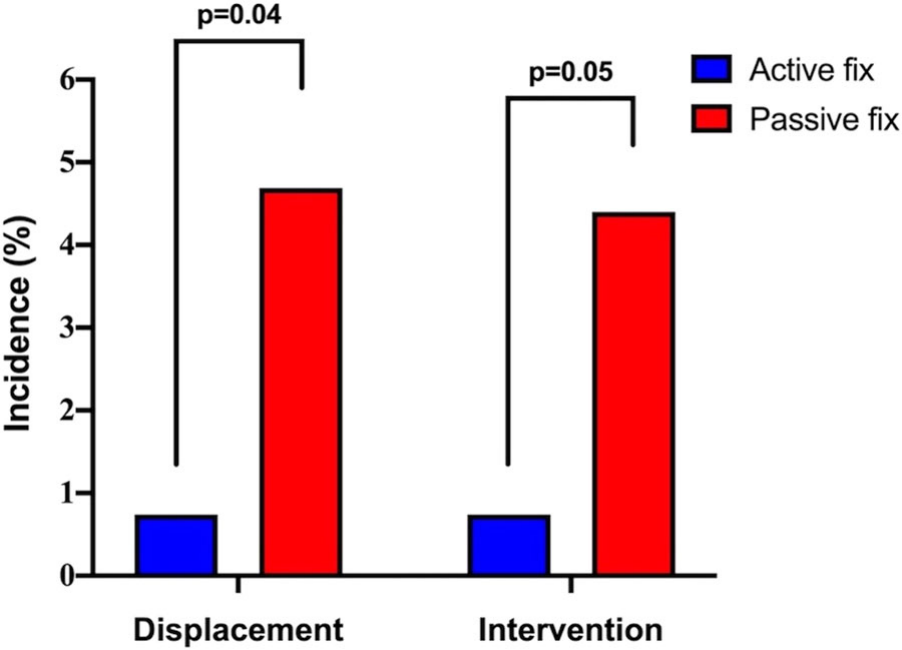
Performance of the active fixation lead: Rates of left ventricular lead displacement and intervention at 6 months post cardiac resynchronization therapy device implantation

The active fixation lead had a significantly lower rate of displacement within 6 months when compared to passive fixation quadripolar leads (1/135, 0.74% vs. 16/341, 4.69%; *p* = .036; Figure 2 and Table 3). There was no significant difference in the rate of displacement of right atrial and right ventricular leads between the two groups. Furthermore, there was no significant difference between the displacement rates of the left ventricular passive fixation leads by the manufacturer (St Jude Medical: 12/305, 3.93%; Medtronic: 3/27, 11.1%; Boston Scientific: 1/9, 11.1%; *p* = .16).

**Table 3:** Complications at 6 months

	Active fix (*n* = 135)	Passive fix (*n* = 341)	*p* value
LV lead displacements	1 (0.74%)	16 (4.69%)	**.04**
Management of LV lead displacements			
Reprogramming	0	1 (0.29%)	1.00
Intervention	1 (0.74%)	15 (4.40%)	**.05**
Phrenic nerve stimulation (PNS)	5 (3.70%)	19 (5.57%)	.49
RA lead displacement	2 (1.48%)	4 (1.17%)	.68
RV lead displacement	3 (2.22%)	6 (1.76%)	.72
Infection requiring revision/explant	0	1 (0.29%)	1.00
Hematoma requiring revision	0	3 (0.88%)	.56
Coronary sinus dissection	4 (2.96%)	14 (4.11%)	.56

*Note*: Data from 476 patients undergoing first‐time cardiac resynchronization therapy device CRT implantation with either a conventional passive fixation LV lead or the active fixation lead.

Abbreviations: CRT, cardiac resynchronization therapy; LV, left ventricular; RA, right atrial.

For patients who experienced a postoperative LV lead displacement, there was limited success in attempting to reprogram the device to overcome the problems associated with lead displacement (successful reprogramming: Active fix = 0/1, Passive fix = 1/16, 6.25%). There was therefore a significantly lower proportion of patients requiring an invasive re‐intervention to revise the lead post‐displacement when an active fixation lead was used (Active fix = 1/135, 0.74% vs. Passive fix = 15/341, 4.40%; *p* = .049; Figure 2 and Table 3).

Rates of phrenic nerve stimulation for the two leads were similar. There was no significant difference between the rates of other procedural complications requiring revision such as wound hematoma and infection (Table 3). Furthermore, the rates of coronary sinus dissection were similar for both groups, and there were no incidences of cardiac tamponade resulting from the placement of an LV lead.

## 4 DISCUSSION

Here we report the first study to directly compare outcomes of a novel active fixation left ventricular lead against conventional passive fixation quadripolar leads for cardiac resynchronization therapy. The active fixation left ventricular lead was associated with reduced rates of lead displacement after cardiac resynchronization therapy. Patients implanted with an active fixation lead were six times less likely to require a revision procedure within 6 months for lead displacement. These findings help to inform the decision‐making process when clinicians select a type of left ventricular lead for cardiac resynchronization therapy.

The introduction of quadripolar leads with additional pacing vectors has reduced lead‐related complications and improved clinical outcomes for patients compared with bipolar LV leads and have now become the lead of choice for most operators.^5^ Despite this, LV lead displacement is a common postprocedure complication with estimated rates ranging from 2% to 10%.^4^, ^14^, ^15^, ^16^ In 2017, a novel quadripolar LV pacing lead with a side‐helix that allows it to be actively fixed to the wall of a target vessel during implantation was introduced to the UK. Initial studies recording the clinical experience with the Attain Stability lead have been promising – a follow‐up study of 82 patients undergoing CRT insertion demonstrated high procedural success rates (98%) with no LV lead displacements after 9 months.^17^ Furthermore, Jackson et al.^10^ have demonstrated a rate of lead displacement of only 0.7% at 6 months. However, before our study, no direct comparison has been made against a control group comprised of patients who underwent insertion of conventional LV quadripolar leads.

In our study, it is notable that both active and passive quadripolar leads had a similarly high success rate for implantation reflecting the improvement in delivery catheters, sub‐selectors, guide wires, stylets, and lead design over the last 20 years. The active fixation lead had a significantly lower pacing threshold and lead impedance which may reflect the better contact obtained from the active fixation mechanism, and/or the fact that all four poles on this lead have steroid eluting electrodes. However, given that pacing energy was similar between active and passive fixation leads, it is unlikely these differences will alter overall battery longevity.

We also demonstrate that there is a significant reduction in the rate of LV lead displacement at 6 months postimplant with the active fixation lead when compared with conventional passive fixation LV leads. Furthermore, reprogramming the device to overcome the issues associated with lead displacement is rarely successful. Out of the 16 patients in the passive fixation group who experienced a lead displacement, 15 were offered a repeat procedure to revise or replace the lead. This represents a significantly higher rate of re‐intervention compared to that when an active fixation lead is inserted in the initial procedure.

In addition to reduced rates of lead displacement, active fixation leads may facilitate LV lead insertion into targeted vein segments and reduce rates of intraoperative displacement, allowing the operator more flexibility in terms of where to position the lead.^18^, ^19^ This is encouraging, since positioning the LV lead at the latest point of activation of the ventricle has been shown to improve clinical outcomes such as mortality and heart failure hospital admission.^15^, ^20^, ^21^ In most cases, targeting the lead to a postero‐lateral, mid‐to‐basal LV segment can achieve pacing at the latest point of activation.^22^

There have previously been concerns expressed about the potential difficulties of extracting chronically implanted active fixation leads.^23^, ^24^ This arose from the analysis of a small number of attempted transvenous extractions of the Medtronic StarFix leads, where powered sheaths and aggressive techniques were required for removal. However, the Attain Stability lead employs a very different mechanism for active fixation into the vessel wall which has been reported to be safe and without procedural complexity in sheep.^25^ Furthermore, there have been successful cases of uncomplicated extraction of the 20066 active fixation bipolar lead, which uses the same technology as the Attain Stability lead.^26^, ^27^ In our study, one active fixation lead required extraction and was done so without complication. Nonetheless, further information about the long‐term extractability of active fixation leads will need to be recorded.

### 4.1 Limitations

This large observational study of 476 patients is based on real‐world data which reflects the routine clinical practice in our center. It is therefore nonrandomized and prone to the effects of possible confounding variables. There was a trend towards older age in the active fixation group. However, it is unlikely that these factors limit the interpretations of our findings given that the key outcome was the rate of lead displacement. Additionally, this is a single‐center study, albeit at a hospital with substantial experience of inserting active fixation leads.

## 5 CONCLUSIONS

The novel active fixation LV lead in our study has a significantly lower rate of lead displacement at 6 months when compared to conventional LV leads. Subsequently, the proportion of patients requiring a repeat procedure to revise the LV lead is also significantly decreased with the active fixation lead.
